# Molecular Mechanisms of Sensorineural Hearing Loss and Development of Inner Ear Therapeutics

**DOI:** 10.3390/ijms22115647

**Published:** 2021-05-26

**Authors:** Srdjan M. Vlajkovic, Peter R. Thorne

**Affiliations:** Department of Physiology and The Eisdell Moore Centre, Faculty of Medical and Health Sciences, The University of Auckland, Private Bag 92019, Auckland 1142, New Zealand; pr.thorne@auckland.ac.nz

The sense of hearing enables us to enjoy sounds and music and engage with other people. The sense of hearing is, however, vulnerable to environmental challenges, such as exposure to noise. Indeed, more than 1.5 billion people experience some decline in hearing ability during their lifetime, of whom at least 430 million will be affected by disabling hearing loss. If not identified and addressed in a timely way, hearing loss can severely reduce the quality of life at various stages, for example by delaying language development, reducing social engagement, compromising economic independence and educational opportunities [1]. The cost of hearing loss has been estimated at more than USD 980 billion annually at a global level [1]. 

Some causes of hearing loss can be prevented, for example from occupational or leisure noise. The World Health Organization (WHO, Geneva, Switzerland) estimates that more than 1 billion young people put themselves at risk of permanent hearing loss by listening to loud music over long periods of time. Mitigating such risks through public health action is essential to addressing hearing loss in the community (WHO, World Report on Hearing, March 2021).

Hearing impairment often results from loss of the sensory hair cells and primary auditory neurons due to disease or trauma to the cochlea of the inner ear. The etiology of sensorineural hearing loss (SNHL) is complex and multifactorial, arising from congenital and acquired causes. Congenital hearing loss commonly manifests as hearing deficits at birth or during early development, while acquired hearing loss is usually sustained in later life from infection, exposure to excessive noise, ototoxic drugs, or the ageing process.

Substantial progress has been made in recent years towards understanding the underlying mechanisms of SNHL and the discovery of novel therapeutic targets to prevent and mitigate the hearing loss. In addition, the link between hearing loss and dementia has been established, with the view that hearing loss prevention may also protect cognitive function.

The aim of this special issue was to advance our understanding of the causes and mechanisms of hearing loss and propose novel strategies to protect and restore hearing. We invited investigators to contribute original research articles and state-of-the-art reviews to address the mechanisms of SNHL caused by cochlear injury or gene mutations, propose new strategies to promote hearing rescue after cochlear injury, and identify novel biomarkers of hearing loss. The collection includes seven original research papers and two reviews from the international groups investigating different aspects of SNHL.

Two articles focused on age-related hearing loss (ARHL). ARHL is the most common sensory disorder among the elderly, characterized by a decline in hearing sensitivity and speech discrimination, delayed central processing of acoustic information, and impaired localization of sound sources [2]. Multiple mechanisms have been proposed for age-related cochlear degeneration, and it appears that both genetic and environmental factors play a role [3]. There are currently no ARHL animal models induced by environmental challenges. Park and colleagues [4] presented a new model of ARHL established by exposing animals to continuous oxidative stress to promote cell ageing. Oxidative stress was induced in mice by intermittent hypoxic conditions, high-fat diet (HFD), or d-galactose injections. Hypoxia had the greatest effect on hearing loss induction, whilst HFD and d-galactose injections induced metabolic changes that promote cell ageing and apoptosis without significant changes in auditory thresholds. When two or more oxidative stress stimuli were combined, hearing loss occurred over a shorter period. The incidence of hearing loss was the highest in triple-exposure conditions than in any single factor model. The authors suggest that this novel animal model can aid in the development of preventative and treatment strategies for ARHL.

Even though approximately one in three people over the age of 65 years suffer from hearing loss, pharmacological therapies for ARHL are still lacking. Previous animal studies have shown that some antioxidants, apoptosis inhibitors, and neuroprotective compounds can delay the onset of ARHL, but none of these compounds successfully completed clinical trials. Szepesy and co-workers [5] propose selegiline, the FDA-approved anti-Parkinsonian drug, as a promising candidate for the treatment of ARHL due to its complex neuroprotective, antioxidant, antiapoptotic, and dopamine release-enhancing effects. In BALB/c mice, selegiline administered in drinking water mitigated the progression of ARHL at frequencies 8–16 kHz. The lack of otoprotective effect in DBA/2J mice indicated strain differences in response to selegiline, and the authors recognize that the otoprotective effect of selegiline depends on the host’s genetic background. 

Noise-induced hearing loss (NIHL) has become a leading occupational health risk in developed countries. NIHL may also result from unsafe recreational, social, and residential noise exposures. People with excessive exposure to noise are frequently the population with a lifestyle of irregular circadian rhythms, which may affect auditory function. The study by Yang and co-workers [6] provides evidence that the disturbed circadian clock induced by exposure to constant light leads to the suppression of circadian clock rhythmicity genes in the cochlea of CBA/CaJ mice without affecting auditory brainstem (ABR) thresholds. However, exposure to high intensity noise in those mice enhanced ABR threshold shifts and increased the loss of outer hair cells and synaptic ribbons relative to the control mice on normal circadian rhythm. This suggests that the dysregulation of the cochlear circadian clock can affect auditory function in the cochlea exposed to acoustic trauma. The study also underlines the importance of the normal circadian clock in the inner ear as a possible mitigation factor in noise-induced cochlear injury.

MicroRNAs are small non-coding single-stranded RNAs that regulate posttranscriptional gene expression. Acute exposure to traumatic noise induces microRNA expression changes not only in the cochlea, but also in central auditory pathways. Park and collaborators [7] demonstrated expression changes of several microRNAs involved in neural plasticity in the cochlear nucleus and inferior colliculus of the rat. Preliminary evidence suggests that these microRNAs may be involved in regulating the MAPK signaling pathway, axon guidance, and the TGF-β signaling pathway. Since miRNAs are stable and can be detected in the blood, they may represent biomarkers for early diagnosis of NIHL. The authors also suggest that the gene therapy involving the transfer of miRNAs to target cells could be used to promote neural plasticity in the central auditory pathways and thus reduce the impact of acoustic injury. 

There is a significant need for the development of effective therapies to prevent permanent cochlear damage and hearing loss after acoustic overexposure. Fok and colleagues [8] identified a novel pharmacological target for the treatment of noise-induced cochlear injury in the post-exposure period. The Regulator of G protein Signaling 4 (RGS4) regulates the activity of G protein-coupled A_1_ adenosine receptors, which confer considerable otoprotection against acoustic trauma and other forms of SNHL such as from cisplatin and aminoglycoside antibiotics. Intratympanic administration of a small molecule RGS4 inhibitor 48 h after exposure to traumatic noise attenuated noise-induced PTS in rats by up to 19 dB, whilst the earlier drug administration (24 h) led to even better preservation of auditory thresholds (up to 32 dB). This was linked to improved survival of sensorineural tissues and afferent synapses in the cochlea. This study demonstrates that intratympanic administration of an RGS4 inhibitor can rescue cochlear injury and hearing loss induced by acoustic overexposure. The authors postulate that the study presents a novel paradigm for the treatment of various forms of SNHL based on regulation of A_1_ receptor signaling. 

On a different note, the paper by Eitelmann and co-workers [9] reported the altered gap junction network topography in mouse models of human hereditary deafness. Mutations in various genes, such as those coding for the voltage-gated calcium channel CaV1.3 or the calcium sensor otoferlin in the inner hair cells of the cochlea, cause hereditary deafness. In genetically modified mouse models lacking these genes, auditory brainstem nuclei are deprived of spontaneous neuronal activity originating in the cochlea, which results in altered neuronal circuitry in auditory brainstem nuclei. This study demonstrates that the altered neuronal circuitry is linked to impaired topography of astrocyte networks in the brainstem. The immunoexpression of astrocytic connexin hemichannels Cx30 and Cx43 increased in the lateral superior olive (LSO) of CaV1.3 and otoferlin knockout mice, respectively. The authors conclude that the spontaneous neuronal activity in the cochlea is crucial for the proper formation of gap junction networks in LSO and point at a critical role of neuron-glia interactions in the auditory brainstem during early postnatal development.

An interesting contribution by Germana and colleagues [10] focused on the development of zebrafish inner ear. The zebrafish is recognized as an important experimental animal model for studying developmental biology and genetics, as well as modelling human disorders including SNHL. The hair cells of the zebrafish inner ear show structural similarity with the sensory hair cells of the mammalian inner ear, but they also have an extraordinary ability to regenerate. This study was undertaken to analyze the cellular localization of brain-derived neurotrophic factor (BDNF) and its receptor TrkB in the inner ear of the zebrafish. BDNF is essential for the development and neuronal plasticity in the brain, but also plays a key role in the regulation and development of the auditory system in mammals. The results of this study demonstrate that the BDNF/TrkB system is present in the sensory cells of the inner ear during the entire life of zebrafish, suggesting that the zebrafish inner ear represents a good model to study the role of neurotrophins in the biology of sensory hair cells. The similarity of the cellular localization of the BDNF/TrkB system in zebrafish and mammals suggests a similar role of this complex in the development and functional maintenance of the inner ear, but may also have implications for the regenerative processes.

The voltage-gated potassium channel KCNQ4 has an essential role in regulating auditory function in the inner ear, by contributing to potassium recycling and maintenance of cochlear homeostasis. Reduced activity of the KCNQ4 channel has been associated with a genetic form of hearing loss, noise-induced hearing loss, and age-related hearing loss [11]. Rim and colleagues presented a comprehensive review of 90 publications looking at the KCNQ4 as a potential therapeutic target for the treatment of hearing loss [11]. In this review, the authors updated the current concepts of the physiological and pathophysiological roles of KCNQ4 in the inner ear and focused on the role of KCNQ4 activators in therapeutic management of different forms of hearing loss. They propose that the simultaneous application of two activators with distinct modes of action may result in synergistic effects and reduced off-target effects. It was also suggested that drug repurposing may be an attractive option for clinical development of KCNQ4 activators as therapies for hearing loss.

Sudden sensorineural hearing loss (SSHL), also known as sudden deafness, is an unexplained, rapid loss of hearing which often affects only one ear and is considered a medical emergency [12]. SSHL can happen to people at any age, but most often affects adults in their late 40s and early 50s. Although about half of people with SSHL recover some or all their hearing spontaneously, usually within one to two weeks from onset, delaying SSHL diagnosis can decrease treatment effectiveness. The comprehensive review by Olex-Zarychta [12] presents hyperbaric oxygen therapy (HBOT) as a medical procedure used as an adjunct therapy for SSHL in addition to oral/intratympanic administration of steroids. In the treatment of SSHL, HBOT is used to reverse the lack of oxygen in the inner ear, which affects the viability of sensorineural tissues. This review focuses on the molecular mechanisms and clinical effectiveness of HBOT in the treatment of SSHL, carefully weighing the risks and benefits of its implementation.

In conclusion, this Special Issue highlights the diverse range of approaches to SNHL, from designing new animal models of ARHL, to the use of microRNAs as biomarkers of cochlear injury and drug repurposing for the therapy of age-related and noise-induced hearing loss. Further investigation into the underlying molecular mechanisms of SNHL and the integration of the novel drug, cell, and gene therapy strategies into controlled clinical studies will permit significant advances in a field where there are currently many unmet needs.

## Data Availability

Not applicable.
